# Shared Decision Making: A Model for Clinical Practice

**DOI:** 10.1007/s11606-012-2077-6

**Published:** 2012-05-23

**Authors:** Glyn Elwyn, Dominick Frosch, Richard Thomson, Natalie Joseph-Williams, Amy Lloyd, Paul Kinnersley, Emma Cording, Dave Tomson, Carole Dodd, Stephen Rollnick, Adrian Edwards, Michael Barry

**Affiliations:** 1 Cochrane Institute of Primary Care and Public Health, Neuadd Meirionydd, Cardiff University, Heath Park, Cardiff, UK CF14 4XN; 2 The Dartmouth Center for Health Care Delivery Science, Dartmouth College, 37 Dewey Field Road, New Hampshire, NH 03755 USA; 3 Department of Health Services Research, Palo Alto Medical Foundation Research Institute, 795 El Camino Real, Palo Alto, CA 94301 USA; 4 Department of Medicine, University of California, Los Angeles, 911 Broxton Avenue, Los Angeles, CA 90024 USA; 5 Institute of Health and Society, Newcastle University, Baddiley-Clark Building, Richardson Road, Newcastle upon Tyne, NE2 4AX UK; 6 Collingwood Health Group, New York Surgery, Brookland Terrace, New York, North Shields, NE29 8EA UK; 7 Clinical Governance & Risk department, Newcastle upon Tyne Hospitals NHS Foundation Trust, Peacock Hall, Royal Victoria Infirmary, Queen Victoria Road, Newcastle upon Tyne, NE1 4LP UK; 8 General Medicine Division, Massachusetts General Hospital, 50 Staniford Street—9th Floor, Boston, MA 0211440 USA; 9 Informed Medical Decisions Foundation, 40 Court Street, Suite 300, Boston, MA 02108 USA

**Keywords:** shared decision making, patient centered care

## Abstract

The principles of shared decision making are well documented but there is a lack of guidance about how to accomplish the approach in routine clinical practice. Our aim here is to translate existing conceptual descriptions into a three-step model that is practical, easy to remember, and can act as a guide to skill development. Achieving shared decision making depends on building a good relationship in the clinical encounter so that information is shared and patients are supported to deliberate and express their preferences and views during the decision making process. To accomplish these tasks, we propose a model of how to do shared decision making that is based on *choice, option* and *decision talk*. The model has three steps: a) introducing choice, b) describing options, often by integrating the use of patient decision support, and c) helping patients explore preferences and make decisions. This model rests on supporting a process of deliberation, and on understanding that decisions should be influenced by exploring and respecting “what matters most” to patients as individuals, and that this exploration in turn depends on them developing informed preferences.

## INTRODUCTION

Sharing decisions, as opposed to clinicians making decisions on behalf of patients, is gaining increasing prominence in health care policy.^1^–^4^ Shared decision making (SDM) has been defined as: ‘an approach where clinicians and patients share the best available evidence when faced with the task of making decisions, and where patients are supported to consider options, to achieve informed preferences”.^2^

The principles of SDM are well documented and the common elements have been summarized.^5^ The earliest mention was in 1982,^6^ but the idea draws on and deepens the principles of patient centered care.^7^,^8^ Others^9^,^10^ provided more detail and this led to a greater focus on the skills required.^11^,^12^ Yet, despite attention to principles and competences, there remains a lack of clear guidance about how to accomplish SDM in routine practice. Our aim is to translate conceptual descriptions into a three-step model that is practical for clinicians. The purpose of this article is to advance a novel, yet pragmatic, proposal about *how to do* SDM in routine settings, in short to integrate good communication skills with the use of patient decision support tools.

## GUIDING ETHICAL PRINCIPLES

The skills of SDM are unlikely to be developed, let alone exhibited, unless the clinician agrees with the guiding ethical principles. At its core, SDM rests on accepting that individual self-determination is a desirable goal and that clinicians need to support patients to achieve this goal, wherever feasible. Self-determination in the context of SDM does not mean that individuals are abandoned. SDM recognizes the need to support autonomy by building good relationships, respecting both individual competence and interdependence on others. These are the key tenets of both self-determination^13^ and relational autonomy.^14^ Self-determination theory is concerned with our intrinsic tendencies to protect and preserve our well-being.^13^ Relational autonomy is the term used to describe the view that we are not entirely free, self-governing agents but that our decisions will always relate to interpersonal relationships and mutual dependencies.^15^ As King and Moulton have noted, these principles extend the concept of informed consent beyond that of simple information transfer towards honoring informed preferences.^16^ We acknowledge that good clinical practice balances these principles with those of beneficence and justice.^17^

However, some healthcare professionals express doubts, saying that patients don’t *want* to be involved in decisions, lack the capacity or ability, might make ‘bad’ decisions, or worry that SDM is just not practical, given constraints such as time pressure. Others claim they are ‘already doing it’, though data from patient experience surveys indicates that this is not generally the case.^18^,^19^ It is therefore clear that the first step for those advocating the uptake of SDM is to ensure that clinicians and others support the underlying rationale.

Before doing so however, we need to note the challenges that clinicians will be navigating. Low health literacy or low numeracy will be barriers to SDM and some patients come from cultural backgrounds that lack a tradition of individuals making autonomous decisions. We cannot therefore emphasize too strongly that SDM has to be built on the core skills of good clinical communication skills, as recognized in many seminal texts,^20^–^23^ including building rapport and structuring the consultations.^24^

## WHY SHARE DECISIONS: BEYOND THE ETHICAL IMPERATIVE

SDM is supported by evidence from 86 randomized trials showing knowledge gain by patients, more confidence in decisions, more active patient involvement, and, in many situations, informed patients elect for more conservative treatment options.^25^ We illustrate the arguments in favor of SDM by providing two hypothetical cases where more than one reasonable treatment option exist—see cases 1 and 2.^12^ They illustrate that informed preferences are an optimal goal because the decisions made will be better understood, based on more accurate expectations about the negative and positive consequences^26^ and more consistent with personal preferences.
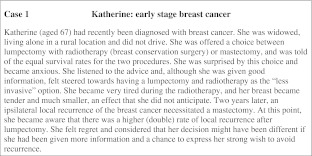
 
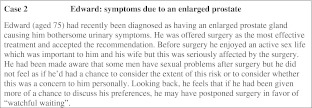


## DOING SHARED DECISION MAKING

We propose that achieving SDM depends on tasks that help *confer agency*, where agency refers to the capacity of individuals to act independently and to make their own free choices.^27^,^28^ SDM aims to confer agency by 1) providing information and 2) supporting the decision making process.

### Providing Information

We help patients participate by providing high quality information. We also need to elicit what patients already know, and whether it is correct. People place different importance on the outcomes associated with different options and have different preferences about the processes and paths that lead to these outcomes. If patients are not informed, they will be unable to assess ‘what it is important to them’, and so establish informed preferences. The first task of SDM is to ensure that individuals are not making decisions when insufficiently informed about key issues, not ‘making decisions in the face of avoidable ignorance’ (Al Mulley, personal communication). Many tools have been designed to help achieve this goal.^29^ Detail about these tools and their effects can be found elsewhere:^11^ in this article we will describe how to deploy them as part of *doing* SDM.

### Supporting Deliberation

**Figure 1.**
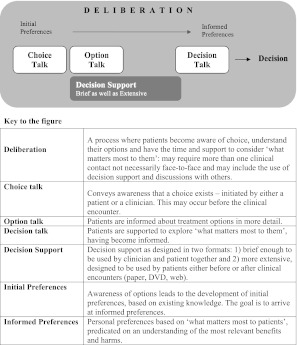
A shared decision making model.

The second task is to support patients to deliberate about their options (see Fig. 1), by exploring their reactions to information. When offered a role in decisions, some patients feel surprised, unsettled by the offer of options and uncertainty about what might be best.^30^ If all responsibility for decision making is transferred to patients they may feel ‘abandoned’.^31^ Some patients initially decline decisional responsibility role, and are wary about participating.^32^

## A MODEL FOR CLINICAL PRACTICE

To accomplish SDM, we propose a three-step model for clinical practice (see Fig. 1). We want to emphasize that this is a simplified model that illustrates the process of moving from initial to informed preferences. We acknowledge that this process also has psychological, social and emotional factors that will influence this deliberation space and that will need to be managed by an effective clinician-patient dialogue, seeking what Epstein has termed a ‘shared mind’.^33^ However, accepting these requirements, we aim for parsimony.

We describe three key steps of SDM for clinical practice, namely: *choice talk, option talk* and *decision talk*, where the clinician supports deliberation throughout the process (Fig. 1 and Boxes 1, 2 and 3). *Choice talk* refers to the step of making sure that patients know that reasonable options are available. *Option talk* refers to providing more detailed information about options and *decision talk* refers to supporting the work of considering preferences and deciding what is best. The model outlines a step-wise process, although it is important to recognize that the model is not prescriptive—clinical interactions are by necessity fluid. Decision support tools provide crucial inputs into this process.
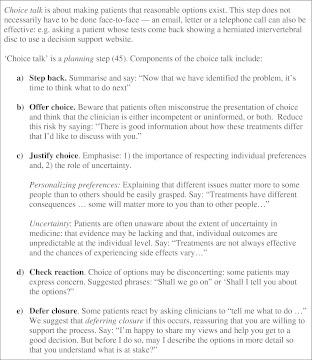
 
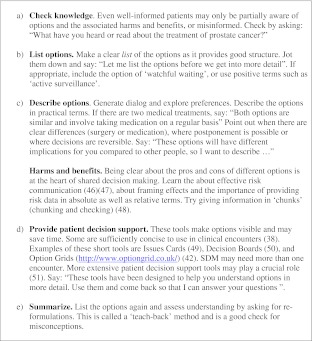
 
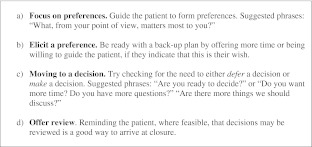


Patients will want time to study new information and to consider their personal preferences, particularly for futures that are unknown to them, to think about outcome states that they have never experienced.^34^,^35^ Deliberation may, in part, be done outside the clinical encounter, although often patients wish to consolidate their views with a trusted clinician. Individuals often want to discuss options with others and it would be best if those involved could potentially use the same information resources (see below). Rapley has referred to this need to talk to others, at different times and places, as a ‘distributed’ deliberation process.^36^ Recognizing this need, and allowing time for it, is a cornerstone for effective SDM .^33^,^37^

The model also includes the use of decision support interventions,^38^ which summarize information in formats that are accessible to patients, using the most up to date evidence about the harms and the benefits.^39^ Some tools also include preference clarification exercises.^39^ Decision support for patients can be in concise formats, such as in brief text or diagrams, and used during encounters to initiate SDM. They can also be extensive: typical of the many tools already developed—booklets, websites, videos, DVDs—to used by patients, their family and friends, outside the encounter, and at different time points^38^—see Box 1, 2 and 3, and synopsis in Box 4.
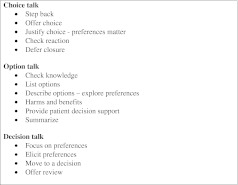


## DELIBERATION

We use the term "deliberation" (see Fig. 1) to describe a process of considering information about the pros and cons of their options, to assess their implications, and to consider a range of possible futures, practical as well as emotional. This ‘deliberation’ space, colored grey in the figure, encompasses the need to work collaboratively with professionals as well as with the wider networks that patients will use.^36^ Deliberation begins as soon as awareness about options develops. The process is iterative and recursive, and the intensity increases after options have been described and understood.

## DISCUSSION

We have proposed a model of how to do SDM in clinical practice (Fig. 1), based on three key steps, namely choice talk (Box 1), option talk (Box 2) and decision talk (Box 3), whilst also being aware that many other people may be contributing. There are implications for training: in our experience the best way to learn these skills is to use simulations, either with colleagues or with trained actors^11^,^40^,^41^ and use brief patient decision support tools.^38^,^42^ There are measurement scales to assess skillfulness,^43^,^44^ although we lack a measure to assess proficiency in risk communication. The use of brief patient decision support tools can catalyze SDM.^38^,^42^

This model builds on the previous work in this field by integrating a number of contributions. It acknowledges the foundations in ethics^9^,^10^ as well as the work that describes the stages and skills required.^5^ However, it was our experience in implementation studies that gave rise to this three-step model that aims to help clinicians integrate SDM and patient decision support into their work.^42^

Many clinicians will push back at the suggestion that yet more has to be accomplished in clinical encounters. We acknowledge this concern and argue that new systems will be required to appropriately reward truly patient centered practice. The introduction of brief decision support interventions can act as a catalyst for a new discourse and help make SDM a practical reality in busy clinics, albeit one that may lead to some patients needing more than one encounter where they can discuss important decisions. We realize that this model is a simplification of a complex, dynamic process, yet its simplicity may help others accomplish and teach shared decision making. That was our goal.
